# Elevated Levels of Serum Tumor Markers CEA and CA15-3 Are Prognostic Parameters for Different Molecular Subtypes of Breast Cancer

**DOI:** 10.1371/journal.pone.0133830

**Published:** 2015-07-24

**Authors:** Yingbo Shao, Xianfu Sun, Yaning He, Chaojun Liu, Hui Liu

**Affiliations:** 1 Department of Breast oncology, The Affiliated Cancer Hospital Of Zhengzhou University, Zhengzhou, China; 2 He’nan Province Tumor Hospital, Zhengzhou, China; University of Nebraska Medical Center, UNITED STATES

## Abstract

**Background & Aims:**

The utility of measuring carcinoembryonic antigen(CEA) and cancer antigen 15-3 (CA15-3) levels in patients with breast cancer remains controversial. The present study aims to investigate the prognostic value of preoperative serum CEA and CA15-3 levels in breast cancer patients.

**Methods:**

Serum preoperative CEA and CA 15-3 concentration levels were measured in a total of 432 breast cancer patients. The association of tumor markers levels with clinicopathological parameters and outcomes were analyzed.

**Results:**

Elevated serum levels of CEA and CA15-3 were identified in 47 (10.9%) and 60(13.9%) patients, respectively. Larger tumor size, advanced axillary lymph nodal and TNM stage exhibited higher proportion of elevated CEA and CA15-3 levels. The elevation of CEA levels was significantly greater in patients with HER2 positive tumors, and the elevation of CA15-3 levels was significantly greater in ER negative breast patients. Univariate and multivariate Cox’s regression analysis revealed that elevated preoperative CEA and CA 15-3 levels were independent prognostic factors for DFS and OS. When considering the combination of both markers levels, patients with both elevated markers presented the worst survival. Independent prognostic significance of elevated preoperative serum CEA and CA15-3 levels were reconfirmed in Luminal B breast cancer.

**Conclusions:**

Preoperative serum levels of CEA and CA15-3 are independent prognostic parameters for breast cancer.

## Introduction

Breast cancer is the most frequently occurring cancer in women and its incidence has been steadily increasing in China[1, 2]. Despite the rising incidence of breast cancer, the survival rates have improved in recent years due to the deep research in biology behavior of breast cancer[3, 4]. However, once treatment failure occurs the quality of life and the survival rate of patients is significantly affected. Therefore, it is essential to identify reliable prognostic factors to guide decision making during the treatment of breast cancer in order to improve prognosis. Along with the traditional pathological factors such as tumor size, tumor grade, lymph node status, molecular markers including hormone receptor status and human epidermal growth factor receptor 2 (HER2) expression[5], serum tumor markers have an important role in screening, early diagnosis of recurrence, and treatment of many malignancies[6, 7]. In breast cancer, carcinoembryonic antigen (CEA) and cancer antigen 15–3 (CA15-3) are the two most widely used serum tumor markers in the clinical fields for more than 30 years.

In recent years, the prognostic value of preoperative CEA and CA15-3 levels in breast cancer has gained much attention. Study has shown that preoperative CEA levels combined with CA15-3 levels may provide useful information for diagnosis and treatment of breast cancer[8–10]. Accordingly, the European Group on Tumor Markers has recommended the CEA and CA15-3 levels be used for assessing prognosis, the early detection of disease progression, and treatment monitoring in breast cancer[11]. However, recently Maric et al. reviewed the role of serum tumor markers in breast cancer and they pointed out conflicting results of its prognostic value[12]. As a result, the American Society of Clinical Oncology (ASCO) guidelines do not currently recommend the use of serum CA 15–3 and CEA for or screening, diagnosis, staging, or routine surveillance of breast cancer patients after primary therapy[13, 14].

Therefore, in the present study, we conducted a retrospective analysis of clinicopathological data of breast cancer patients, to explore the relationships between preoperative serum CEA, CA15-3 levels and clinicopathological parameters, as well as the prognostic value of these two serum biomarkers in breast cancer.

## Materials and Methods

### Study Population and Follow-up

From January 2002 to December 2004, serum CEA and CA 15–3 in a total of 432 patients who were treated for stage I–III invasive breast cancer at The Affiliated Cancer Hospital of Zhengzhou University were investigated. Inclusion criteria were: female; invasive breast cancer; underwent mastectomy or breast-conserving surgery; CEA and CA15-3 levels were determined before surgery; tumor completely removed by surgery with pathologic evaluation; appropriate adjuvant chemotherapy, adjuvant radiotherapy and endocrine therapy administered based on international guidelines; complete results of estrogen receptor (ER), progesterone receptor (PR), HER2, Ki-67, and histologic grade. Exclusion criteria were: stage IV breast cancer; carcinoma in situ; neoadjuvant chemotherapy cases. This study was reported according to the Reporting Recommendations for Tumor Marker Prognostic Studies (REMARK) criteria[15].

TNM staging was based on the sixth American Joint Committee on Cancer criteria. ER and PR positive were defined as tumors with >1% nuclear-stained cells. HER2-positivity was indicated by a 3+ or 2+ score from the immunohistochemical evaluation, and was confirmed using a fluorescence in situ hybridization (FISH) test for HER2. A cut-off point of 14% was used for Ki-67 staining. The molecular subtypes were classified into four groups as follows: Luminal A (ER+and/or PR+, HER2-,Ki-67<14%); Luminal B (ER+ and/or PR+, HER2+ and/or Ki-67 ≥14%); HER2 positive (ER- and PR-, HER2+); and triple-negative (ER- and PR-,HER2-) according to the molecular subtype consensus of the St. Gallen International Expert Consensus on the Primary Therapy of Early Breast Cancer 2011[16].

With the day pathological diagnosis was performed considered as the first day of follow-up, clinical follow-up was carried out every 6 to 12 months, which included recording patient’s history, physical examination, laboratory tests of CEA, CA 15–3, chest radiography, breast and abdominopelvic ultrasonography, and bone scans. Disease-free survival (DFS) was defined to be from the time of surgery to the locoregional recurrence, distant metastasis, and death before recurrence. Overall survival (OS) was defined to be from the time of surgery to death from any cause. Locoregional recurrence was defined as pathologically confirmed relapse on the chest wall, supra- and infraclavicular fossa, axillary area, or internal mammary region. Distant metastasis was confirmed using medical imaging method, and pathology assessment if needed.

### Ethics Statement

This study was carried out in accordance with the ethical guidelines of the 1975 Declaration of Helsinki and was approved by the ethics committee of the Affiliated Cancer Hospital Of Zhengzhou University. Written informed consent was obtained from every patient for the use of the serum samples and medical records for research purposes.

### Sample Collection and tumor marker analysis

All serum samples were collected in the morning. Blood collected without anticoagulant was centrifuged at 1600 × g for 10 min at 4°C one hour after collection and transferred into tubes and kept at −80°C for further experimentation. Serum CEA and CA15-3 levels were determined using an automatic electrochemistry luminescence immunoassay system (ROCHE E170; Roche, Germany). The cut-off values of CEA and CA15-3 were 5.0 ng/mL and 25 U/mL, respectively, and the value was considered positive or negative for the marker if the level was above or below the cut-off value, respectively.

### Statistical analysis

The χ^2^ and Fisher’s exact probability tests were used to analyze the differences between proportions. DFS and OS were estimated using the Kaplan–Meier method and compared using the log-rank test. Independent prognostic factors for DFS and OS were identified by multivariate Cox’s proportional hazard analysis. All the statistical analyses were performed with SPSS 17.0 (SPSS, Chicago, IL, USA) software. A P value < 0.05 was considered to be statistically significant.

## Results

### Clinicopathological characteristics of patients

The study enrolled a total of 432 patients who were treated for stage I–III invasive breast cancer at The Affiliated Cancer Hospital Of Zhengzhou University. The clinicopathological characteristics of the patients are shown in Table 1. The median age of the study population was 50 years (range 16–78 years). Stage Ⅰ, Ⅱ and Ⅲ breast cancer accounted for 25%, 46.5%, and 28.5% respectively. Among all the cases, Luminal B(246,56.9%) accounted for the largest proportion, 68 (15.8%) were classified as Luminal A, 44 (10.2%) as HER2 positive, and 74 (17.1%) as triple-negative. Parts of original clinicopathological characteristics of patients are packaged in S1 Fig.

**Table 1:** Clinicopathological characteristic of subjects and correlation between serum CA 15–3 and CEA level and clinicopathological factors.

	n	CEA			CA15-3		
		Normal (%)	Elevated (%)	*P*	Normal (%)	Elevated (%)	*P*
**Age**							
** ≤35 years**	**43(10.0)**	**41(95.3)**	**2(4.7)**	**0.167**	**34(79.1)**	**9(20.9)**	**0.159**
** >35 years**	**389(90.0)**	**344(88.4)**	**45(11.6)**		**338(86.9)**	**51(13.1)**	
**Tumor size**							
** T1**	**136(31.5)**	**129(94.9)**	**7(5.1)**	**<0.001** *	**124(91.2)**	**12(8.8)**	**<0.001** *
** T2**	**243(56.2)**	**223(91.8)**	**20(8.2)**		**212(87.2)**	**31(12.8)**	
** T3**	**31(7.2)**	**20(64.5)**	**11(35.5)**		**22(71.0)**	**9(29.0)**	
** T4**	**22(5.1)**	**13(59.1)**	**9(40.9)**		**14(63.6)**	**8(36.4)**	
**Nodal status**							
** N0**	**269(62.3)**	**247(91.8)**	**22(8.2)**	**0.019** *	**237(88.1)**	**32(11.9)**	**0.005** *
** N1**	**92(21.3)**	**82(89.1)**	**10(10.9)**		**81(88.0)**	**11(12.0)**	
** N2**	**45(10.4)**	**36(80.0)**	**9(20.0)**		**33(73.3)**	**12(26.7)**	
** N3**	**26(6.0)**	**20(76.9)**	**6(23.1)**		**21(80.8)**	**5(19.2)**	
**TNM stage**							
** Ⅰ**	**108(25.0)**	**103(95.4)**	**5(4.6)**	**0.023** *	**102(94.4)**	**6(5.6)**	**0.007** *
** Ⅱ**	**201(46.5)**	**178(88.6)**	**23(11.4)**		**167(83.1)**	**34(16.9)**	
** Ⅲ**	**123(28.5)**	**104(84.6)**	**19(15.4)**		**102(82.9)**	**21(17.1)**	
**ER**							
** Negative**	**131(30.3)**	**115(87.8)**	**16(12.2)**	**0.557**	**102(77.9)**	**29(22.1)**	**0.001** *
** Positive**	**301(69.7)**	**270(89.7)**	**31(10.3)**		**270(89.7)**	**31(10.3)**	
**PR**							
** Negative**	**162(37.5)**	**151(93.2)**	**11(6.8)**	**0.034** *	**136(84.0)**	**26(16.0)**	**0.315**
** Positive**	**270(62.5)**	**234(86.7)**	**36(13.3)**		**236(87.4)**	**34(12.6)**	
**HER2**							
** Negative**	**295(68.3)**	**272(92.2)**	**23(7.8)**	**0.003** *	**252(85.4)**	**43(14.6)**	**0.544**
** Positive**	**137 (31.7)**	**113(82.5)**	**24(17.5)**		**120(87.6)**	**17(12.4)**	
**Ki-67 (%)**							
** <14%**	**63(14.8)**	**60(95.2)**	**3(4.8)**	**0.092**	**57(90.5)**	**6(9.5)**	**0.278**
** ≥14%**	**369(85.2)**	**325(88.1)**	**44(11.9)**		**315(85.4)**	**54(14.6)**	
**Molecular subtype**							
** Luminal A**	**68(15.8)**	**63(94.1)**	**5(5.9)**	**0.016** *	**62(91.2)**	**6(8.8)**	**0.012** *
** Luminal B**	**246(56.9)**	**210 (85.4)**	**36(14.6)**		**218(88.6)**	**28(11.4)**	
** Her-2 positive**	**44(10.2)**	**40(90.9)**	**4(9.1)**		**32(72.7)**	**12(27.3)**	
** TNBC**	**74(17.1)**	**72(97.3)**	**2(2.7)**		**60(81.1)**	**14(18.9)**	

TNM tumor-node-metastasis, ER estrogen receptor, PR progesterone receptor, HER2 human epidermal growth factor receptor 2, CA 15–3 cancer antigen 15–3, CEA carcinoembryonic angigen, TNBC triple-negative breast cancer.

*P < 0.05 indicates a significant difference.

### Relationship between serum CEA and CA15-3 and clinicopathological characteristics of patients

Elevated serum levels of CEA and CA15-3 were identified in 47 (10.9%) and 60(13.9%) patients, respectively. The correlation between serum CEA and CA15-3 levels and clinicopathological characteristics of patients are shown in Table 1. Both CEA and CA15-3 levels were correlated with the size of the primary tumor and axillary lymph node status, larger tumor size, advanced axillary lymph nodal and TNM stage exhibited higher proportion of elevated serum tumor markers. The elevation of CEA levels was significantly greater in patients with HER2 positive tumors (7.8% vs 17.5% p = 0.003) but not with ER expressions, however, the elevation of CA15-3 levels was significantly greater in ER negative breast patients (22.1% vs 10.3% p = 0.001). Increased CEA and CA15-3 levels were not associated with age and Ki-67. The elevation of CEA levels was significantly greater in patients with HER2 positive tumors (14.6% in Luminal B and 9.1% in HER2 positive tumors) than in patients with the other two subtypes (5.9% in Luminal A and 2.7% in triple-negative tumors). While, the elevation of CA15-3 levels was significantly greater in patients with ER negative tumors (27.3% in HER2 positive and 18.9% in triple-negative tumors) than in patients with the other two subtypes (8.8% in Luminal A and 11.4% in Luminal B tumors).

### Prognostic value of serum CEA and CA15-3 levels in breast cancer patients

**Fig 1:**
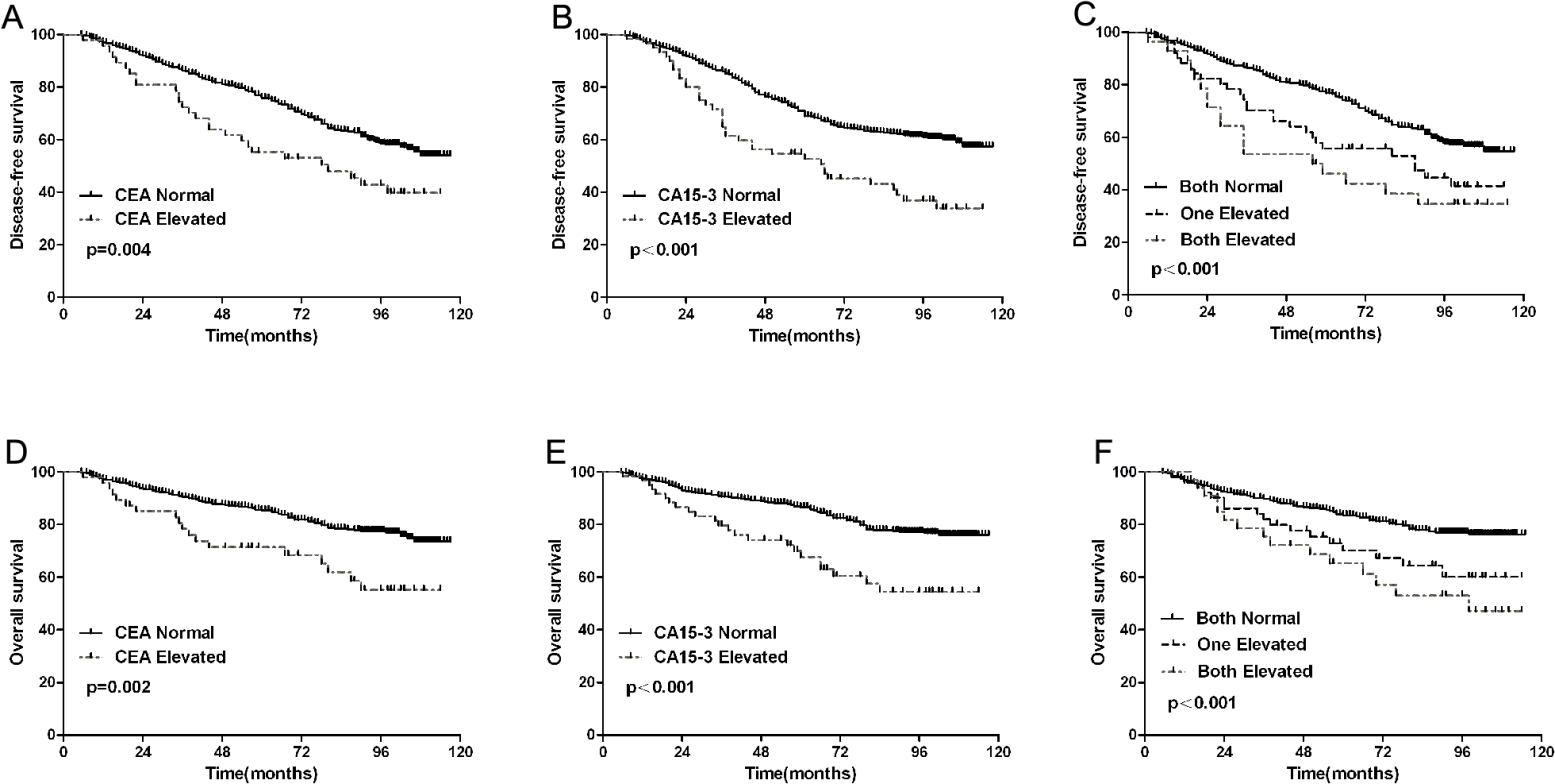
Kaplan-Meier survival curves of patients with normal or elevated CEA and CA15-3 levels. Disease-free survival (DFS) according to carcinoembryonic antigen (CEA) (A) and cancer antigen 15–3 (CA15-3) (B). Overall survival (OS) according to CEA(D) and CA15-3(E). DFS according to the combination of both marker levels(C) and OS according to the combination of both marker levels (F).

In our study population of 432 subjects of breast cancer, the median follow-up time was 80 months (range from 6 months to 118 months), elevated CEA and CA 15–3 levels were significantly associated with DFS and OS of breast cancer patients (Table 2 and Fig 1). Univariate analysis showed that the 5-year DFS and OS of CEA-elevated vs. CEA-normal patients were 57.4% vs. 77.9% (P = 0.004), 88.8% vs. 74.4% (P = 0.002), respectively. The 5-year DFS and OS of CA15-3-elevated vs. CA15-3-normal patients were 74.4% vs. 88.8% (P = 0.002), 71.7% vs. 90.8% (P<0.001), respectively. Clinicopathological factors such as age, tumor size, node metastasis, ER status, HER2 status and molecular subtype also had independent prognostic power.

**Table 2:** Univariate analysis of prognostic factors for survival time of breast cancer patients.

	n	DFS		OS	
		5-year (%)	*P*	5-year (%)	*P*
**Age**					
** ≤35 years**	**43**	**67.4**	**0.094**	**74.4**	**0.011** *
** >35 years**	**389**	**78.7**		**88.2**	
**Tumor size**					
** T1**	**136**	**73.5**	**<0.001** *	**83.1**	**0.021** *
** T2**	**243**	**63.4**		**79.4**	
** T3**	**31**	**51.6**		**71.0**	
** T4**	**22**	**40.9**		**54.5**	
**Nodal status**					
** N0**	**269**	**86.6**	**<0.001** *	**90.0**	**<0.001** *
** N1**	**92**	**71.7**		**85.9**	
** N2**	**45**	**53.3**		**71.1**	
** N3**	**26**	**46.2**		**65.4**	
**TNM stage**					
** Ⅰ**	**108**	**80.6**	**<0.001** *	**85.2**	**<0.001** *
** Ⅱ**	**201**	**68.7**		**77.3**	
** Ⅲ**	**123**	**48.0**		**61.0**	
**ER**					
** Negative**	**131**	**64.9**	**0.141**	**76.3**	**0.013** *
** Positive**	**301**	**71.7**		**86.0**	
**PR**					
** Negative**	**162**	**73.5**	**0.627**	**71.6**	**0.001** *
** Positive**	**270**	**75.6**		**84.8**	
**HER2**					
** Negative**	**295**	**60.0**	**<0.001** *	**75.9**	**0.011** *
** Positive**	**135**	**80.0**		**86.0**	
**Ki-67 (%)**					
** <14%**	**63**	**73.0**	**0.914**	**82.5**	**0.739**
** ≥14%**	**369**	**72.3**		**80.7**	
**Molecular subtype**					
** Luminal A**	**68**	**79.4**	**0.001** *	**83.8**	**<0.001** *
** Luminal B**	**246**	**71.5**		**82.1**	
** Her-2 positive**	**44**	**59.1**		**63.6**	
** TNBC**	**74**	**51.4**		**59.5**	
**CEA**					
** Normal**	**385**	**77.9**	**0.004** *	**88.8**	**0.002** *
** Elevated**	**47**	**57.4**		**74.4**	
**CA15-3**					
** Normal**	**372**	**76.6**	**<0.001** *	**90.8**	**<0.001** *
** Elevated**	**60**	**53,3**		**71.7**	

TNM tumor-node-metastasis, ER estrogen receptor, PR progesterone receptor, HER2 human epidermal growth factor receptor 2, CA 15–3 cancer antigen 15–3, CEA carcinoembryonic angigen, TNBC triple-negative breast cancer, DFS disease-free survival, OS overall survival.

*P < 0.05 indicates a significant difference.

Multivariate Cox’s regression analysis according to tumor size, nodal status, stage, HER2 status, and serum tumor markers revealed that elevated serum CEA and CA 15–3 levels were independent prognostic factors for DFS and OS (Table 3). 28 patients exhibited both elevation of CEA and CA 15–3 levels, either CEA and CA 15–3 was elevated in 51 patients. When considering the combination of both markers levels, Kaplan–Meier survival curve revealed that patients with normal levels of both the two tumor markers showed the best DFS and OS, those with both elevated markers presented the worst survival.

**Table 3:** Multivariate Cox’s regression analysis according to tumor size,nodal status, stage, HER2 status, and serum tumor markers.

	DFS			OS		
	HR	95%CI	*P*	HR	95%CI	*P*
**Tumor size**						
** T1-T2**						
** T3-T4**	**1.831**	**1.262–2.165**	**0.014** *	**1.640**	**1.020–1.771**	**0.162**
**Nodal status**						
** N0-N1**						
** N2-N3**	**2.156**	**1.588–3.260**	**<0.001** *	**2.327**	**1.667–3.304**	**<0.001** *
**TNM stage**						
** Ⅰ+Ⅱ**						
** Ⅲ**	**1.842**	**1.270–2.345**	**0.022** *	**1.979**	**1.346–2.661**	**0.012** *
**HER2**						
** Negative**						
** Positive**	**1.417**	**1.235–1.817**	**0.019** *	**1.765**	**1.320–1.974**	**0.006** *
**CEA**						
** Normal**						
** Elevated**	**1.832**	**1.281–2.623**	**<0.001** *	**2.042**	**1.332–2.853**	**<0.001** *
**CA15-3**						
** Normal**						
** Elevated**	**2.012**	**1.393–2.648**	**<0.001** *	**2.319**	**1.066–2.979**	**<0.001** *

TNM tumor-node-metastasis, HER2 human epidermal growth factor receptor 2, CA 15–3 cancer antigen 15–3, CEA carcinoembryonic angigen, DFS disease-free survival; OS overall survival, HR, Hazard ratio, CI, Confidence interval.

*P < 0.05 indicates a significant difference.

**Fig 2:**
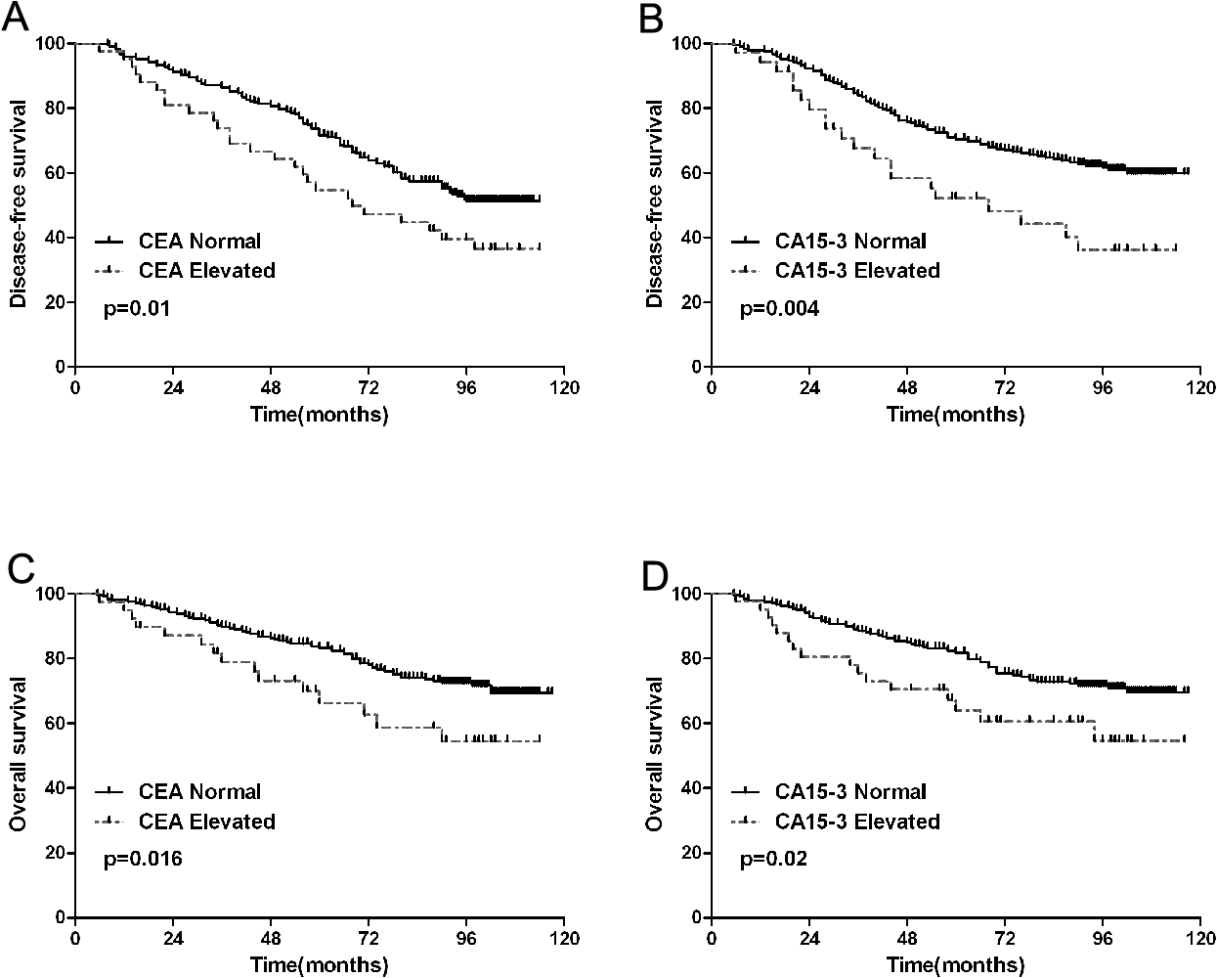
Kaplan-Meier survival curves of Luminal B breast patients with normal or elevated CEA and CA15-3 levels. DFS according to CEA(A) and CA15-3(B), OS according to CEA(C) and CA15-3(D).

Luminal B(246,56.9%) accounted for the largest proportion in the present study, prognostic value of serum CEA and CA15-3 levels in Luminal B breast cancer patients was further analyzed. Both CEA and CA 15–3 levels were independent prognostic factors for DFS and OS in Luminal B breast cancer patients (Fig 2).

## Discussion

The prognostic value of preoperative serum tumor marker CEA and CA 15–3 was evaluated in our present study including 432 patients, the results showed that preoperative serum CEA and CA15-3 levels were independent factors affecting prognosis.

The utility of measuring CEA and CA15-3 levels in patients with breast cancer remains controversial. European Group on Tumor Markers has recommended the CEA and CA15-3 levels be used for assessing prognosis, the early detection of disease progression, and treatment monitoring in breast cancer[11]. The American Society of Clinical Oncology (ASCO) and the National Comprehensive Cancer Network (NCCN) guidelines do not currently recommend the use of serum CA 15–3 and CEA for breast cancer screening and directing treatment[14]. On the one hand, this may partly due to the conflicting conclusions of different researches[8, 9, 12, 17–19], on the other hand, the low positive rate of serum tumor markers is also the possible reason. Studies by Wu et.al found that CEA and CA15-3 levels were elevated in 7.2% and 12.3% breast cancer cases, respectively[9]. In our present study, elevated serum levels of CEA and CA15-3 were identified in 47 (10.9%) and 60(13.9%) patients, respectively.

The incidence of breast cancer has been steadily increasing in the last two decades, however, due to the early detection and increased use of more effective systemic therapy, the survival rates of breast cancer have improved in recent years, and early breast cancer accounted for a large proportion. Previous researches demonstrated that the CEA and CA15-3 levels are associated with tumor burden indicators including tumor size and lymph node status[17, 20] and patients with locally advanced breast cancer exhibit significantly higher levels of CEA and CA 15–3[21, 22]. The present study also demonstrated that higher levels of CEA and CA 15–3 are more common in patients larger tumor size, advanced axillary lymph nodal and TNM stage. As expected, with the increase of early breast cancer patients, the prevalence of abnormal serum CA 15–3 and CEA decreased. However, this does not mean that their clinical value is also low.

Since elevated levels of CA 15–3 and CEA are related to the tumor burden and higher levels may indicate an increased likelihood of systemic metastases. Studies by Lee et.al showed that elevated tumor marker levels are more frequently observed in metastatic breast cancer patients than in primary breast cancer, and patients who had elevated tumor marker levels before surgery also showed more frequent elevation at recurrence [23]. Since markers are relatively easy and inexpensive to measure, regular measurement of serum tumor marker levels could provide useful information for earlier detection of recurrence [24, 25].

In terms of serum tumor markers association with survival outcome, our present study confirmed the independent prognostic value of elevated serum CEA and CA 15–3 levels for breast cancer, elevated CA 15–3 and CEA levels were accompanied by worse DFS and OS. And furthermore, when considering the combination of both markers levels, Kaplan–Meier survival curve revealed that patients with both elevated markers have the worst prognosis. At the same time, traditional clinical and pathological features of breast cancer, including nodal status, TNM stage, and internal molecular parameter, including Her-2 status, molecular subtype are also important prognostic indicators for breast cancer, multivariate Cox’s regression analysis demonstrated that nodal status, stage, HER2 status were independent prognostic factors for DFS and OS In addition to CA 15–3 and CEA. However, there is no perfect indicator which contains all the prognostic information, on the basis of the traditional indicators, serum CEA and CA 15–3 provides additional valuable prognostic information for breast cancer.

Reports on the correlation between CEA and CA15-3 and molecular subtypes, however, are rare. In our present study, the elevation of CEA levels was significantly greater in HER2 positive patients, and the elevation of CA15-3 levels was significantly greater in ER negative breast patients. Simultaneously, the elevation rates of CEA and CA15-3 levels in different subtypes of breast cancer is also different, which may be explained in part by the different biological behaviors of different molecular subtypes. Luminal B(246,56.9%) accounted for the largest proportion in the present study. Luminal B breast cancer is recognized as having an aggressive clinical behavior, it’s molecular characterization, clinical management have not been clarified clearly[26–28]. So far, there is no effective prognostic indicator for Luminal B breast cancer. In the present study, prognostic value of serum CEA and CA15-3 levels in Luminal B breast cancer patients was further analyzed. Both CEA and CA 15–3 levels were independent predictors for DFS and OS in Luminal B breast cancer patients. The utility of these serum biomarkers may be served as effective prognostic indicators for Luminal B breast cancer patients. Further researches are needed to determine the effectiveness of these serum biomarkers in formulating treatment strategies in clinical practice.

In conclusion, our present study confirmed the independent prognostic value of elevated serum CEA and CA 15–3 levels for breast cancer, when considering one or the combination of both markers. Elevated preoperative serum tumor markers could be useful in determining the risk of recurrence and metastasis of breast cancer after operation, and further analyses, including a prospective study design, are needed to clarify the utility of these serum biomarkers in treatment decision-making area for breast cancer.

## Supporting Information

S1 Fig Parts of original clinicopathological data of patients. (RAR)
